# Effect of integrated intervention to prevent child drowning in rural areas of Guangdong, China: a cluster randomized controlled trial

**DOI:** 10.1093/tropej/fmad012

**Published:** 2023-04-05

**Authors:** Ruilin Meng, Haofeng Xu, Mingqu Zhang, Pengpeng Ye, Zhishan Zhou, Xuhao Zhu, Xingru Li, Lifeng Lin

**Affiliations:** Guangdong Centre for Disease Control and Prevention, Institute of Chronic Noncommunicable Disease Prevention and Control, 160 Qunxian Road, Panyu district, Guangzhou 511430, China; Guangdong Centre for Disease Control and Prevention, Institute of Chronic Noncommunicable Disease Prevention and Control, 160 Qunxian Road, Panyu district, Guangzhou 511430, China; Qingyuan City Centre for Disease Control and Prevention,Institute of Chronic Noncommunicable Disease Prevention and Control, 6 Kangle Road, Qingcheng district, Qingyuan 511518, China; Chinese Centre for Disease Control and Prevention, National Chronic Disease Center, 155 Changbai Road, Changping district, Beijing 102206, China; Qingxin District Centre for Disease Control and Prevention, Institute of Chronic Noncommunicable Disease Prevention and Control, ′15 Fuqian Road, Taihe Town,′′ Qingyuan 511899,′ China; Qingyuan City Centre for Disease Control and Prevention,Institute of Chronic Noncommunicable Disease Prevention and Control, 6 Kangle Road, Qingcheng district, Qingyuan 511518, China; Qingxin District Centre for Disease Control and Prevention, Institute of Chronic Noncommunicable Disease Prevention and Control, ′15 Fuqian Road, Taihe Town,′′ Qingyuan 511899,′ China; Guangdong Centre for Disease Control and Prevention, Institute of Chronic Noncommunicable Disease Prevention and Control, 160 Qunxian Road, Panyu district, Guangzhou 511430, China

**Keywords:** non-fatal drowning, integrated intervention, children, rural areas, China

## Abstract

**Background:**

Drowning is the leading cause of death for children under the age of 15 years in Guangdong Province, China. This serious public health issue also exists in low- and middle-income countries (LMICs), which have few value-integrated intervention programs. The current study presents an integrated intervention project that aims to explore an effective pattern of prevention for child drowning in rural areas and feasibility to perform in other LMICs.

**Methods:**

We conducted a cluster randomized controlled trial by comparing the incidence of non-fatal drowning among children in two groups in rural areas of southern China. We recruited the participants in two phases and reached a total of 10 687 students from 23 schools at two towns in Guangdong Province, China. At the first and second phases, 8966 and 1721 students were recruited, respectively.

**Results:**

The final evaluation questionnaires were collected after 18 months of integrated intervention, where we obtained 9791 data from Grades 3–9. The incidence of non-fatal drowning between the intervention and control groups after intervention did not differ significantly from the baseline according to the total number of students, male students, female students and Grades 6–9 [0.81; 95% confidence interval (CI): [0.66, 1.00]; *p *=* *0.05, 1.17; 95% CI: [0.90, 1.51]; *p *=* *0.25, 1.40; 95% CI: [0.97, 2.02]; *p *=* *0.07 and 0.97; 95% CI: [0.70, 1.34]; *p *=* *0.86], except for Grades 3–5 (1.36; 95% CI: [1.02, 1.82]; *p *=* *0.037). The study observed a significantly positive benefit of awareness and risk behaviours of non-fatal drowning between the intervention and control groups (0.27, 95% CI: [0.21, 0.33]; *p *=* *0.00, −0.16; 95% CI: [−0.24, −0.08]; *p *=* *0.00).

**Conclusions:**

The integrated intervention exerted a significant impact on the prevention and management of child non-fatal drowning, especially in rural areas.

## INTRODUCTION

The disease burden of child drowning remains at a high level, such that it is among the top 10 leading causes of death among children and young people worldwide [1]. China, India, Pakistan and Bangladesh account for 51.2% of the total death rate due to drowning worldwide in 2017 [2]. The mortality rate in China related to drowning is the fourth highest among G20 countries [3].

The rates of child drowning exhibited a continuing decline in high-income countries (HICs). Prevention strategies in view of the peculiar characteristics of child drowning included promoting multisectoral collaboration, strengthening public awareness of drowning through communication, establishing a national water safety plan and conducting research on the prevention of drowning in HICs [1, 4–9].

In low- and middle-income countries (LMICs), different prevention strategies may be required according to area due to the significant variation in the characteristics of drowning. Many integral health promotion programs were developed with specific objectives, which included improving health care, developing health behaviours and monitoring the number of accidents. Scholars suggested educating guardians to master supervision skills as a key measure in the prevention of child injury [10, 11]. In Bangladesh, several intervention projects achieved successful results through childcare centres and swimming lessons, which were highly cost-effective strategies [12–14]. Exploring the Szpilman clinical score in the paediatric emergency department, which was based on the features of prognostic factors, was one of the important strategies for prevention drowning [15]. In Brazil, disparate compensation policies were established for the reduction of injury [16].

In China, drowning is the leading cause of death in children aged 1–14 years [17, 18], although the disease burden of drowning of children declined significantly from 1990 to 2015 [19]. Therefore, it remains a serious health issue in China. In Guangdong Province, which is located in the south of China, drowning is the top leading cause of death among children aged 1–14 years with a mortality rate of 3.86 per 100 000 [20]. Rural areas are especially severe at the risk of drowning for children, where the mortality rate is 2.42 times that of urban areas (rural and urban areas: 5.08 and 2.10 per 100,000, respectively) [21].

In China, three health education programs on the prevention of child drowning for children or parents were implemented in rural areas in Guangdong, Jiangsu, Zhejiang Province, which reduced morbidity/mortality related to drowning in childhood [22–24]. After the implementation of a program for an integrated intervention for drowning that targeted floating children in an urban area in Zhejiang Province, the incidence of non-fatal drowning in an experiment group was significantly lower than that prior to the intervention [25].

Intervention strategies, such as enhancing the quality of caregivers through education, which were effective in other countries, may not be feasible in rural areas in China due to regional differences [26, 27]. Local governments in Guangdong, Jiangsu and Zhejiang implemented several effective health education services for children, which aimed to single out risk factors in rural areas [22–24].

The study, which used a repeated-measure intervention-control design, conducted a 3-year program to provide intervention to prevent drowning for rural students. The integrated intervention project aimed to explore an effective pattern of prevention for child drowning in rural areas and to examine the feasibility of implementing the program to other LMICs.

## METHODS

Based on the mortality surveillance data for Guangdong Province, we conducted a study in two rural townships in Qingyuan City, namely, Jintan and Longjing, which feature abundant nature and man-made bodies of water and high mortality rates due to drowning. We used the cluster random sampling strategy to assign the two townships into intervention (Jintan) and control (Longjing) groups through simple randomization (i.e. a coin toss). The study recruited students from 23 schools (18 elementary schools and 5 middle schools) in the two towns. The students were cluster randomized into the intervention or control group. The target populations were all students in Grades 3–8 (8–18 years old) at baseline and Grades 3–9 at the final evaluation (18 months after the intervention).

### Participant and public involvement

The development of the research questionnaire and outcome measures was informed by a study by Ma, *et al.* [28] on drowning prevention in Guangdong Province.

The study recruited 8966 and 1721 students for two phases, respectively, for a total of 10 687. At the second phase of the study, 896 (544 and 352 students from the intervention and control groups, respectively) were excluded after graduation (in China, students in elementary, middle and high schools generally do not change schools except after graduation). Informed consent was obtained from all subjects (parents or legal guardians provided consent for subjects aged less than 18 years).

The intervention group received the integrated intervention, and the control group received general health education prior to the final evaluation. At the start of the program, we collected 8390 responses to the self-reported questionnaire out of 8966 students in Grades 3–8 in the two towns. Out of the 8390 responses, 73 were excluded due to missing information. Prior to the end of the program, the study collected a total of 9791 responses from students in Grades 3–9 for both groups for the final evaluation survey. Out of 9791 responses, the study further excluded 34 due to missing information (Table 1).

**Table 1.** Basic characteristics of the two towns

	Intervention township (*n*)	Control township (*n*)
Baseline		
No. schools	10	13
No. classes	126	108
No. students	5157	3160
Final evaluation		
No. schools	10	13
No. classes	128	108
No. students	6094	3663

Institutions involved in the study will also help with the dissemination of the findings: we intend to demonstrate the results to local authorities to enforce the incorporation of the intervention in the future and engage the development of drowning policies.

Institutions did not assess the burden of the intervention, because the project was funded by the National Centre for Chronic Non-Communicable Disease Control and Prevention, Health Commission of Guangdong Province.

### Integrated intervention

This program was implemented from July 2014 to December 2016. The intervention group received the integrated intervention, which included systematic health education, community actions, policies promotion strategies and environmental improvement (Table 2).

**Table 2.** Components of integrated intervention and measurement times

Systematic health education	Sponsors	Implementation
All students		
** **Class meeting of prevent drowning	Teachers	Once a week for 5 weeks
** **Program of knowledge of drowning and relative risk factors	CDC and Schools	Once
** **Program: looking for dangerous water in the eyes of children	CDC and Schools	Once
** **Health manuals, bulletin, lectures	CDC	Every month for 10 months
** **Skills training: water safety training, self-rescuing skill, rescuing skill	CDC and Red cross Society	Once
Teachers		
** **Program of knowledge of drowning and relative risk factors	CDC and Schools	Once
** **A competition of lecture in the class meeting	Schools	Once (including five lectures)
** **Skills training: water safety training, self-rescuing skill, rescuing skill	CDC and Red cross Society	Once
** **Health manuals, bulletin, lectures	CDC	Every month for 10 months
Guardians		
** **Program of knowledge of drowning and relative risk factors	CDC and Schools	Once
** **Health manuals, bulletin	CDC	Once
** **Program of supervision education	CDC and Schools	Once
Community action		
** **The village committee organized supervision of the water	CDC and local government	Once, for 2 months in the summer
** **The village committee organized supervision of children	CDC and local government	Once, for 2 months in the summer
Policies promotion strategies		
** **The local government issued a document	Local government	Once
Environmental improvement		
** **The local government set up bamboo fence or barrier by the pond	Local government	Once

Furthermore, all students and their families and teachers in the intervention group underwent health education. For students, the Guangdong Provincial Centre for Disease Control and Prevention and the Red Cross Society of Guangdong Province and its volunteers provided regular systematic health education (knowledge about drowning, prevention skills about water safety training, self-rescue skills, rescue skills through health manuals, bulletins and lectures). The guardians of students in the intervention group (including new students in Grade 3) received health manuals, bulletins and lessons in water safety, high-risk factors and supervision skills. Demonstrations of drowning prevention skills (self-rescue and rescue skills) were performed in all schools for the intervention group for the majority of teachers (class teachers, gym teachers and Principals of Security).

Community actions were conducted by village committees, which organized villagers to establish river patrol parties and childcare teams to monitor the water and supervise students. The local government issued a document that outlined the responsibilities of relevant institutions in implementing environmental improvement and the rescue of drowning children. These strategies included surrounding the water with bamboo fences or barriers and building self-latching gates for houses.

**Figure 1:**
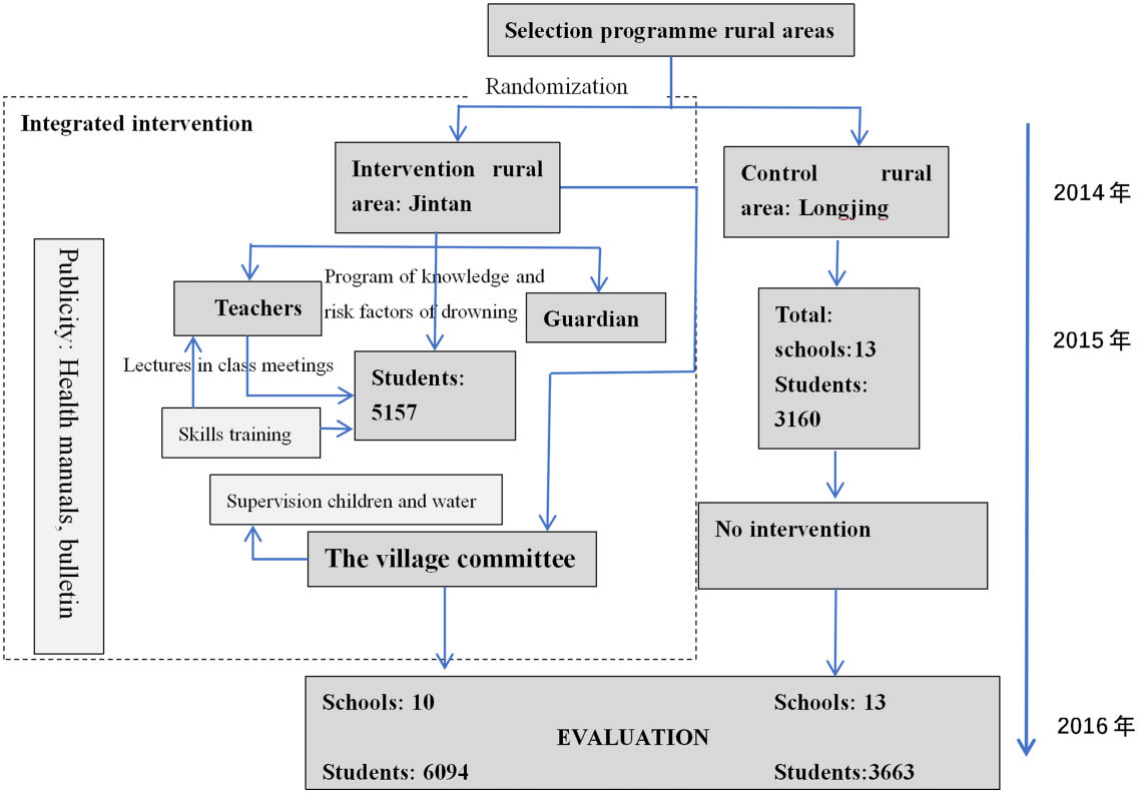
Model for the implementation of the integrated intervention for the prevention of children drowning in Guangdong Province, China, using a cluster randomized controlled trial.

For the control group, general safety education manuals were distributed to the participants (including their guardians and teachers). After the project, the control group also received the same intervention for drowning prevention (Figure 1).

### Data collection and analysis

The study conducted baseline and final evaluation surveys for both groups. The design of the questionnaire was based on a previous study conducted on child drowning prevention [28]. The surveys collected information about the number of non-fatal drowning events in the previous year, potential drowning-related risk factors, general information (e.g. age, gender and grade), level of education of parents, relationships between family members, level of swimming skills, perception on drowning, high-risk behaviours (e.g. swimming in ponds and playing beside rivers), environmental conditions (e.g. distance from school to open water, distance from home to open water and the presence of any open body of water on the way to school) and disease burden related to drowning (cost of treatment after drowning events). The investigators would help Grade 3–6 students to finish the questionnaires and check all the questionnaires after they were finished by all the students at each survey to ensure quality (e.g. integrity of information, avoiding contradictory information).

The study used the same questionnaire with the same definition of drowning for both groups in a double-blind method by investigators. According to the World Health Organization, drowning is the process of experiencing respiratory impairment from submersion/immersion in liquid. The outcomes are classified as death, morbidity and no morbidity [1]. The total score for knowledge about drowning was equal to the number of correct responses (correct = 1, wrong = 0 and total score = 7). The same rules are applied to calculate the total score for risk behaviours related to drowning (total score = 4; Table 3).

**Table 3.** The information in the questionnaire

Questions	Correct answers = 1	Wrong answers = 0
Knowledge		
Drowning is among the top causes of death for children.	Correct = 1	Wrong = 0
Can drowning be prevented among children?	Yes = 1	No = 0
Which diseases do you think cause easy drowning?	Cardiovascular = 1	Dermatology; tympanitis; myopic = 0
What is the best way to escape when your driving car falls in the water?	Holding on and opening the door when the air pressure in the car equal to outside = 1	Braking out the car windows and rush out at once; waiting for rescue with all car lights on; do not know how to do = 0
Losing conscious after 2 minutes of drowning, how many minutes are needed when nervous system is damaged?	4–6 min = 1	7–10; 11–15; have no idea about this = 0
Which is the main function of plastic swimming ring?	Having fun = 1	Lifesaving; learning to swim; have no ideas about this = 0
During cardiopulmonary resuscitation, what is the ratio of times of cardiac compression and artificial respiration?	30:2 = 1	15:2; 5:1; have no idea about this = 0
Risk behaviors		
Did you swim in the open water without your guardian’s company during past 12 months?	No = 1	More than three times; 1–2 times/month; 1–2 times/3 months; 1–2 times/12 months = 0
Did you fish alone in the open water during past 12 months?	No = 1	More than three times; 1–2 times/month; 1–2 times/3 months; 1–2 times/12 months = 0
Did you play with your partners around the pool (or open water or swimming pool) during past 12 months?	No = 1	More than three times; 1–2 times/month; 1–2 times/3 months; 1–2 times/12 months = 0
Did you dive in the open water unknown depth during past 12 months?	No = 1	More than three times; 1–2 times/month; 1–2 times/3 months; 1–2 times/12 months = 0

The sample size was calculated based on the intracluster correlation coefficient (ICC: the ratio of between-cluster variance to between- and within-cluster variances), drowning incidence rate and the expected effect size. The rate of incident of non-fatal drowning among children was estimated at 10% for Grades 3–9 [29, 30]. The study set the expected reduction in the rate of incidence of drowning among children to 50%, and an ICC value of 0.011, which was based on the study on non-fatal drowning among children, allowed 5% loss to follow-up students.

The two townships were randomly assigned to the intervention and control groups. The study employed univariate analysis using *t*-tests for continuous variables and normal distribution (knowledge and behaviour) [31] and chi-squared analysis for categorical variables to examine baseline risk factors and their association with group assignment (a two-sided type-I error of 5%).

The study analysed the differences in the incidence of drowning, knowledge and high-risk behaviours between the intervention and control groups at the end of project using the difference-in-differences (DID) method. Analyses were performed using SPSS V.21.0 and SAS version 9.2.

## RESULTS

The study obtained a total of 8317 questionnaires from students in Grades 3–8 at baseline; the average ages [standard deviation (SD)] were 12.5 (1.9; *n* = 5157) and 12.7 (1.8; *n* = 3160) years for the intervention and control groups, respectively. The number of male students in the intervention group (51.6%) was less than that of the control group (54.0%; *χ*^2^ = 4.67, *p *<* *0.05; Table 4).

**Table 4.** Baseline characteristics of students in the two groups

	Intervention group	Control group	*p*-Value
Age (years), mean (SD)	12.5 (1.9)	12.7 (1.8)	0.001 (*F* = 10.812)
Gender			0.031
** **Boys (%)	2660 (51.6%)	1707 (54.0%)	
** **Girls (%)	2497 (48.4%)	1453 (46.0%)	
Incidence of non-fatal drowning			
** **Total students	669/5157 (13.0%)	344/3160 (10.9%)	0.005
** **Boys	421/2660 (15.8%)	228/1707 (13.4%)	0.025
** **Girls	248/2497 (9.9%)	116/1453 (8.0%)	0.025
** **Grades 3–5	447/2609 (17.1%)	212/1458 (14.5%)	0.031
** **Grades 6–8	221/2546 (8.7%)	132/1698 (7.8%)	0.295
Awareness (score)			
** **Total students	2.8 ± 1.2	2.8 ± 1.3	0.156 (*F* = 2.015)
** **Boys	2.8 ± 1.2	2.8 ± 1.3	0.482 (*F* = 0.494)
** **Girls	2.9 ± 1.4	2.8 ± 1.2	0.246 (*F* = 1.347)
Risk behaviors (score)			
** **Total students	2.9 ± 1.3	3.0 ± 1.3	0.002 (*F* = 10.044)
** **Boys	2.6 ± 1.4	2.7 ± 1.4	0.013 (*F* = 6.122)
** **Girls	3.3 ± 1.1	3.4 ± 1.0	0.007 (*F* = 7.396)

For the final evaluation survey, the study obtained a total of 9757 responses from students in Grades 3–9 (control group: 3663; intervention group: 6094). The average ages (SD) of intervention and control groups were 12.2 (1.9) and 12.2 (2.0) years, respectively. The study observed no difference in the basic demographic characteristics between the two groups (*χ*^2^ = 0.64, *p *>* *0.05).

### Incidence of non-fatal drowning

At baseline, the intervention group displayed a higher incidence rate for non-fatal drowning in terms of the total number of students, male students, female students and Grades 3–5 than that of the control group (*p *=* *0.025, *p *=* *0.025 and *p *=* *0.031, respectively) but not for Grades 6–8 (*p *=* *0.295).

Table 5 presents the primary outcome measures for the two groups at the 18-month follow-up. The difference in the incidence of non-fatal drowning between the intervention and control groups after intervention did not differ significantly from that at baseline for the total number of students, male students, female students and Grades 6–9 [0.81; 95% confidence interval (CI): [0.66, 1.00]; *p *=* *0.05, 1.17; 95% CI: [0.90, 1.51]; *p *=* *0.25, 1.40; 95% CI: [0.97, 2.02]; *p *=* *0.07 and 0.97; 95% CI: [0.70, 1.34]; *p *=* *0.86], except Grades 3–5 (1.36; 95% CI: [1.02, 1.82]; *p *=* *0.037, Table 5).

**Table 5.** The primary outcome at 18-month follow-up of the integrated intervention

	Intervention group	Control group	Difference [95% CI]	*p*-Value
Incidence of non-fatal drowning				
Total students	7.0%	7.0%	0.81 [0.66, 1.00]	0.05
** **Boys	9.7%	9.3%	1.17 [0.90, 1.51]	0.25
** **Girls	4.2%	4.6%	1.40 [0.97, 2.02]	0.07
** **Grades 3–5	8.0%	10.6%	1.36 [1.02, 1.82]	0.037
** **Grades 6–9	6.7%	5.9%	0.97 [0.70, 1.34]	0.86
Awareness (score)				
Total students	3.2 ± 1.5	2.7 ± 1.3	0.27 [0.21, 0.33]	0.00
** **Boys	3.1 ± 1.5	2.6 ± 1.4	0.21 [0.19, 0.36]	0.00
** **Girls	3.3 ± 1.3	2.8 ± 1.3	0.26 [0.17, 0.35]	0.00
Risk behaviors (score)				
Total students	3.1 ± 1.3	3.0 ± 1.3	−0.16 [−0.24, −0.08]	0.00
** **Boys	3.1 ± 1.2	3.0 ± 1.3	−0.16 [−0.27, −0.03]	0.01
** **Girls	3.1 ± 1.2	3.1 ± 1.4	−0.17 [−0.26, −0.08]	0.00

### Changes in awareness and risk behaviours related to non-fatal drowning

By the end of the study, awareness and risk behaviours displayed positive benefits (0.27; 95% CI: [0.21, 0.33]; *p *=* *0.00, −0.16; 95% CI: [−0.24, −0.08]; *p *=* *0.00, respectively.). The scores for awareness and risk behaviours related to non-fatal drowning were 3.2 ± 1.5 and 3.1 ± 1.3 for the intervention group and 2.7 ± 1.3 and 3.0 ± 1.3 for the control group. The same results were awareness (0.21; 95% CI: [0.19, 0.36]; *p *=* *0.00, 0.26; 95% CI: [0.17, 0.35]; *p *=* *0.00) and behaviours (−0.16; 95% CI: [−0.27, −0.03]; *p *=* *0.01, −0.17; 95% CI: [−0.26, −0.08]; *p *=* *0.00, respectively; Table 5) for male and female students.

## DISCUSSION

In China, drowning mortality in 2017 was higher than the global average despite the substantial decline in the disease burden of drowning from 1990 to 2015 [2, 19]. Thus, reducing the mortality of child drowning in China became a challenge as the drowning rates for children aged 1–4 and 5–14 years were the highest and second-highest among G20 countries, respectively [3]. Intervention studies on drowning among children, especially in rural areas, were few in China; hence, the objective of this project was to explore the patterns of drowning prevention among children in rural areas.

Analysis suggested that integrated intervention could reduce the incidence of non-fatal drowning among younger students. Alternatively, the results implied significant positive changes in knowledge and behaviour according to the total number of students in the intervention group. The risk factors reported by other studies [32, 33] were similar to those of a previous study [29, 30]. The study identified age, gender, poverty, level of education, rural residence and environment as risk factors for child drowning. The reason for the positive influence of younger students on the incidence of drowning after the intervention was that helping them develop positive habits was easier than that for older students. The promotion of the social environment, i.e. social health education may provide the public with high levels of knowledge about children safety, should also strengthen the positive benefits for both groups. The study found no difference in the reduction of the incidence of non-fatal drowning between two groups in terms of the total number of students, male students, female students and Grades 6–9. We consider that the results were related to the short intervention time.

According to a review, the current study obtained results similar to those of other drowning prevention studies in HICs, which were found to be effective only on intermediary outcomes (increased levels of drowning skills and knowledge). However, the study was unable to find any impact of the reduction of the number of drowning events among the target population [34–37]. In LMICs, including China, studies reported reduced incidences of non-fatal and fatal drowning, whereas the level of knowledge was increased after the intervention for drowning prevention [25, 38, 39].

The risk factors for child drowning in rural areas included open water, low levels of knowledge and skills and unsupervised of children access to water. Hence, the important topics of drowning prevention studies are improving the home environment and natural bodies of water and the development of knowledge and skills against drowning among the target population [31, 40–44] Furthermore, drowning prevention among children was considered a synthetic project, which requires multisectoral collaboration in the issuance of policies, improvement of the environment (surrounding pools with bamboo fences and barriers) and the dissemination of knowledge [45]. For the empowerment of subjects, the health educational intervention performed in this study is frequently used as a major communication tool to help elicit positive effects on the rate of decline in drowning and increase in the levels of knowledge and skills of drowning among students, their caregivers, teachers and local governments [11, 44]. The study observes that the low levels of knowledge and risk behaviours related to drowning among students at baseline improved significantly through the health education intervention.

Among childhood injury events, drowning ranked first as the cause of death in Guangdong Province, China [21]. Based on the epidemiological features of injury among children, the urgent need for intervention for drowning prevention was evident. As multiple factors can cause child drowning, the integrated intervention should be considered more effective than intervention measures that only target one factor. To the best of our knowledge, studies in LMICs that focused on integrated prevention projects for drowning prevention among students were relatively few. The current study was a large-scale one conducted in rural areas in China to assess the effectiveness of the integrated intervention for the prevention of non-fatal drowning among students in Grades 3–9.

This study has its limitations. First, data were based on self-reports, which may induce recall bias. Second, the results were impacted when the control group was exposed to the community education for child safety.

## CONCLUSION

The program of the integrated intervention for drowning prevention of non-fatal drowning among younger students has increased awareness and reduced risk behaviours associated with child drowning. The study explored an effective pattern for the integrated prevention of child drowning in LMICs.

## Data Availability

The datasets used and/or analysed during the current study are available from the corresponding author on reasonable request.
